# Admissions to a Low-Resource Neonatal Unit in Malawi Using a Mobile App and Dashboard: A 1-Year Digital Perinatal Outcome Audit

**DOI:** 10.3389/fdgth.2021.761128

**Published:** 2021-12-23

**Authors:** Yamikani Mgusha, Deliwe Bernadette Nkhoma, Msandeni Chiume, Beatrice Gundo, Rodwell Gundo, Farah Shair, Tim Hull-Bailey, Monica Lakhanpaul, Fabianna Lorencatto, Michelle Heys, Caroline Crehan

**Affiliations:** ^1^Paediatric Department, Kamuzu Central Hospital, Lilongwe, Malawi; ^2^Parent and Child Health Initiative, Lilongwe, Malawi; ^3^Medical and Surgical Nursing Department, Kamuzu College of Nursing, University of Malawi, Lilongwe, Malawi; ^4^Royal College of Science, Imperial College London, London, United Kingdom; ^5^Population Policy and Practice Department, Great Ormond Street Institute of Child Health, University College London, London, United Kingdom; ^6^Centre for Behaviour Change, University College London, London, United Kingdom; ^7^Specialist Children's and Young People's Services, East London National Health Service Foundation Trust, London, United Kingdom

**Keywords:** infant, newborn, low income population, mobile health, health informatics application, data dashboard, clinical audit, digital health

## Abstract

**Introduction:** Understanding the extent and cause of high neonatal deaths rates in Sub-Saharan Africa is a challenge, especially in the presence of poor-quality and inaccurate data. The NeoTree digital data capture and quality improvement system has been live at Kamuzu Central Hospital, Neonatal Unit, Malawi, since April 2019.

**Objective:** To describe patterns of admissions and outcomes in babies admitted to a Malawian neonatal unit over a 1-year period *via* a prototype data dashboard.

**Methods:** Data were collected prospectively at the point of care, using the NeoTree app, which includes digital admission and outcome forms containing embedded clinical decision and management support and education in newborn care according to evidence-based guidelines. Data were exported and visualised using Microsoft Power BI. Descriptive and inferential analysis statistics were executed using R.

**Results:** Data collected *via* NeoTree were 100% for all mandatory fields and, on average, 96% complete across all fields. Coverage of admissions, discharges, and deaths was 97, 99, and 91%, respectively, when compared with the ward logbook. A total of 2,732 neonates were admitted and 2,413 (88.3%) had an electronic outcome recorded: 1,899 (78.7%) were discharged alive, 12 (0.5%) were referred to another hospital, 10 (0.4%) absconded, and 492 (20%) babies died. The overall case fatality rate (CFR) was 204/1,000 admissions. Babies who were premature, low birth weight, out born, or hypothermic on admission, and had significantly higher CFR. Lead causes of death were prematurity with respiratory distress (*n* = 252, 51%), neonatal sepsis (*n* = 116, 23%), and neonatal encephalopathy (*n* = 80, 16%). The most common perceived modifiable factors in death were inadequate monitoring of vital signs and suboptimal management of sepsis. Two hundred and two (8.1%) neonates were HIV exposed, of whom a third [59 (29.2%)] did not receive prophylactic nevirapine, hence vulnerable to vertical infection.

**Conclusion:** A digital data capture and quality improvement system was successfully deployed in a low resource neonatal unit with high (1 in 5) mortality rates providing and visualising reliable, timely, and complete data describing patterns, risk factors, and modifiable causes of newborn mortality. Key targets for quality improvement were identified. Future research will explore the impact of the NeoTree on quality of care and newborn survival.

## Introduction

Neonatal mortality was estimated at 2.5 million globally in 2017, representing 47% of all deaths, under the age of five (1). The majority (90%) of neonatal deaths occur in sub-Saharan Africa and South Asia, regions which have a neonatal mortality rate of 27 and 26 per 1,000 live births, respectively (1). Malawi is a resource-poor country in sub-Saharan Africa, consistently ranked among the least developed in the world (2). Approximately, 62% of its 16.7 million inhabitants live below the international poverty line of $1.25 per day (3). Despite other challenges, various initiatives have improved the health of children in Malawi in the last few decades (4). These include the Emergency Triage and Treatment program (5), Safe motherhood programs (6), Helping Babies Breath (7), national Malawian neonatal guidelines “Care of the infant Newborn,” (8) and Integrated Management of Childhood Illness (4). The country reached its fourth World Health Organisation (WHO) Millennium Development Goal to reduce childhood mortality by two-thirds in 2013 (9). Despite these gains, Malawi still has high neonatal mortality rates of 27 per 1,000 live births (10). The three main causes of neonatal deaths in Malawi, like other low-resource settings, are prematurity, neonatal encephalopathy, and neonatal sepsis (11). Preventable or modifiable perinatal factors frequently contribute to the leading causes of neonatal mortality in low-resource settings. This is in contrast to high resource settings where the main causes of neonatal mortality are congenital abnormalities, which are less modifiable (12).

Many neonatal deaths are unaccounted for in low resource settings due to lack of timely, accurate, and complete data. Previous studies have highlighted key contributory factors such as insufficient equipment (e.g., paper supplies), absence of computerised patient record keeping, difficulties in reliable record keeping, and poor documentation (4, 13, 14). Perinatal audit or “death audit” is a hospital level process of accounting for neonatal deaths in maternal and child health services, and is one of the WHO standards for improving quality of care for small and sick newborns in health facilities (15). Audit can be defined as a continuous quality improvement process that seeks to improve patient care and outcomes through systematic review of care against well-defined standards (16). Perinatal mortality audit is defined by the WHO as the process of capturing information on the number and causes of stillbirths and neonatal deaths, and then identifying specific cases for systematic and critical analysis of the quality of care received. Ideally, this should be conducted in a no-blame, interdisciplinary setting, with a view to improving the care provided for all mothers and babies (17). Perinatal audits also aim to assess avoidable factors contributing to death, modifiable factors as well as substandard factors (18), and in Malawi, “modifiable factors” around deaths are recorded routinely on Ministry of Health death reporting forms (Appendix A).

Previous published audits in low-resource settings have included a paediatric death audit at a tertiary hospital, Kamuzu Central Hospital (KCH), Malawi, demonstrating that 48 (5.8%) medical paper files were missing, and, furthermore, within available medical files there were clinically significant missing components such as absence of recording of a baby's vital signs on admission (4). A prospective perinatal audit in Kenya, which used electronic data capture from paper records and audit and feedback cycles to support a common inpatient neonatal data platform, concluded that audit and feedback might be more effective if based on data that are valid and timely (14). A point-of-care, prospective electronic audit was conducted at Zomba Central Hospital (ZCH), another tertiary hospital in Malawi in 2016, using an early version of the NeoTree app. This showed promising results, achieving more than 90% completeness of data, and 70% coverage of admissions in a phased implementation where the NeoTree was done in addition to paper forms (19) (coverage here is defined as the proportion of admissions captured electronically compared to usual paper documentation). A larger 1-year audit of neonatal mortality rates has been conducted more recently at KCH; however, this utilised a data clerk to enter paper records into a database after admissions had taken place and did not report monthly trends in detail (20). Since the ZCH pilot (21), the NeoTree has been iteratively co-developed, implemented, and evaluated at Sally Mugabe Central Hospital, Zimbabwe (from October 2018) (22, 23), KCH (from April 2019) (21, 24) and in Chinhoyi Provincial Hospital, Zimbabwe (from November 2020). During the implementation at KCH, a dashboard prototype was developed to visualise data on a screen on the neonatal unit and provide a monthly slide deck of morbidity and mortality (M&M) data (25). Development and maintenance of the NeoTree android app have been a collaborative, end-user, and data-driven effort, employing open-source code while maintaining patient confidentiality, therefore aligning with the principles of digital development (26). For example it was designed with the end-users as proposed by digital development principle 1. It has also been designed with input and support from the ministry of health, building on existing data collection processes and working towards integration with the DHIS in accordance with the Malawi eHealth strategy (27).

Thus far, a large electronic neonatal audit collected on bedside tablet devices by health professionals at the point-of-care, and reported on a data dashboard, has not yet been conducted in a low-resource neonatal unit. We, therefore, report a 1-year, digital neonatal outcome audit *via* a data dashboard at KCH, aiming to identify patterns in neonatal admissions and outcomes, describe monthly trends relating to significant events, and report coverage by an electronic point of care app, using usual paper- based data collection processes as the presumed gold standard.

## Materials and Methods

### Study Setting

In Malawi, health care services are provided by public (no charge) and private (for-profit and not-for- profit) sectors. Public sector services include all health facilities under government ministries and district, town, and city councils. The private for-profit sector includes private hospitals and clinics as well as traditional healers. Private not-for-profit services comprise non-governmental organisations, statutory corporations, companies, and religious institutions, for example, the Christian Health Association of Malawi (CHAM), which provides ~29% of all health services (12). Broadly, all services are organised into three levels of health care: primary (health centres), secondary (district/CHAM hospitals), and tertiary (central hospitals) linked to each other through an established referral system.

The hospital setting for the study was “Ethel Mutharika Neonatal Unit” at KCH, Lilongwe, which is one of four public tertiary referral hospitals in Malawi. It serves the central region of the country and a population of ~5 million people (1). The neonatal unit has a capacity of 80 beds and admits five to 10 sick neonates each day, including sick or vulnerable neonates delivered within the hospital, referrals from other facilities, as well as sick or vulnerable babies who were delivered at home. There are 17 permanent neonatal nurses on the rota with three to five nurses covering day shifts and two nurses at night. There are two clinical officers and a paediatric specialist who is the overall in charge of the unit. The neonatal unit provides medical interventions and treatments including continuous positive airways pressure, phototherapy, and nasal cannula oxygen as well as intravenous and oral medications. Neonatal surgical procedures are undertaken for babies with gastroschisis, exomphalos, necrotising enterocolitis, and some other congenital anomalies. The neonatal unit comprises four separate areas, namely: “high risk” for unstable neonates requiring respiratory support, “low risk” for stable infants requiring feeding support, “isolation” for patients requiring isolation and admitted from home, and fourthly, a ward for Kangaroo Mother Care. Admission forms for new patients admitted to the unit are completed by nurses.

### Study Design

**Figure 1:**
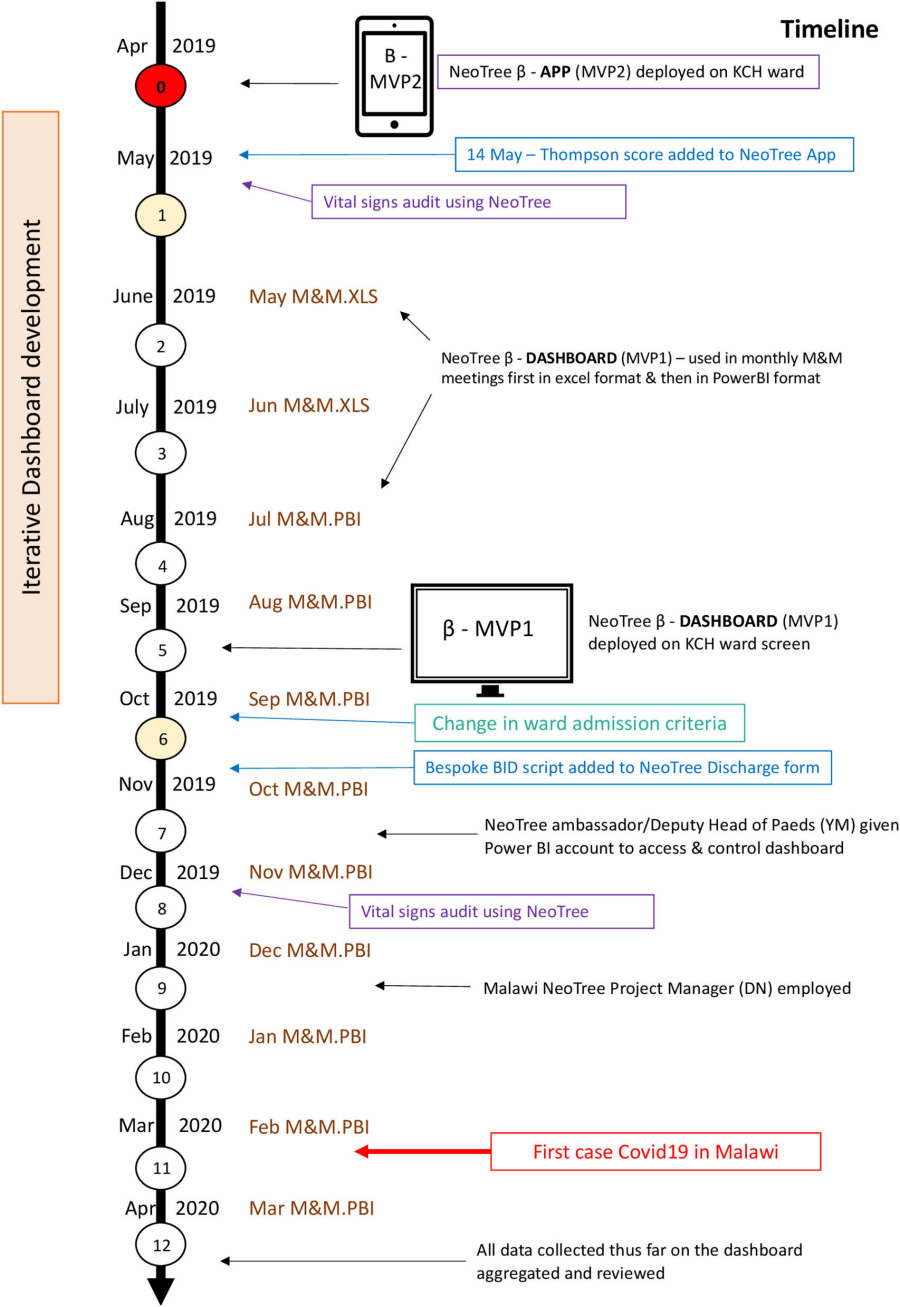
The study timeline. MVP, minimum viable product; KCH, Kamuzu Central Hospital; M&M, morbidity and mortality; XLS, microsoft excel; PBI, microsoft power BI (buisiness intelligence).

A descriptive prospective, point-of-care, electronic audit of admissions to the KCH Neonatal unit over a period of 1 year, May 2019–April 2020 (see Timeline—Figure 1).

### Data Collection

Data were collected prospectively by nursing staff at the point of care using electronic admission and outcome forms within an app “NeoTree” on tablet devices, completely replacing the previous Ministry of Health (MOH) paper forms, as part of usual care. Both forms contain embedded clinical decision and management support, education, and training in neonatal care according to national and international guidelines: “Care of the Infant Newborn” (COIN) and WHO guidelines, respectively (28).

#### Data Collected on Admission

Digital admission forms on the NeoTree App include all required MOH fields under the following section headings: *emergency triage, vital signs, patient demographics, examination findings, symptom review*, and *maternal antenatal, labour, and birth history*. In the emergency triage and vital signs sections, the stabilisation of any sick neonate with abnormal vital signs is supported in real time by an emergency decision support algorithm and management support. Both the admission and discharge forms have simple field validation applied at data entry level. For example, number fields are limited to a plausible range and the correct number of digits and decimal places. Presenting complaint/referral reason is recorded near the beginning of an admission whilst provisional diagnosis is entered at the end of the form by the admitting nurse Both presenting complaint and provisional diagnosis fields have multiple choice lists where more than one problem or diagnosis can be selected. Currently the NeoTree does not use national diagnostic codes. But this is planned, and the current list of diagnoses were reviewed and agreed by the local team, in line with national and international guidelines (15, 28). Healthcare professionals are provided with management support guidelines according to the diagnoses selected at the end of the admission process. The Thompson score for neonatal encephalopathy (validated in low resource settings) (29), was added to the admission form on 14th May, 2019, for at-risk infants >36 weeks' gestation early in the audit timeline, according to expert opinion (30) and co-development with the local team at KCH.

#### Data Collected on Discharge/Death

Digital outcome forms include multiple choice lists of causes of death for neonates that have died and clinician diagnoses for live discharges, where each patient can have more than one cause of death or diagnosis. Healthcare professionals are also asked document modifiable risk factors associated with deaths in a free text field.

#### Description of NeoTree System

**Figure 2:**
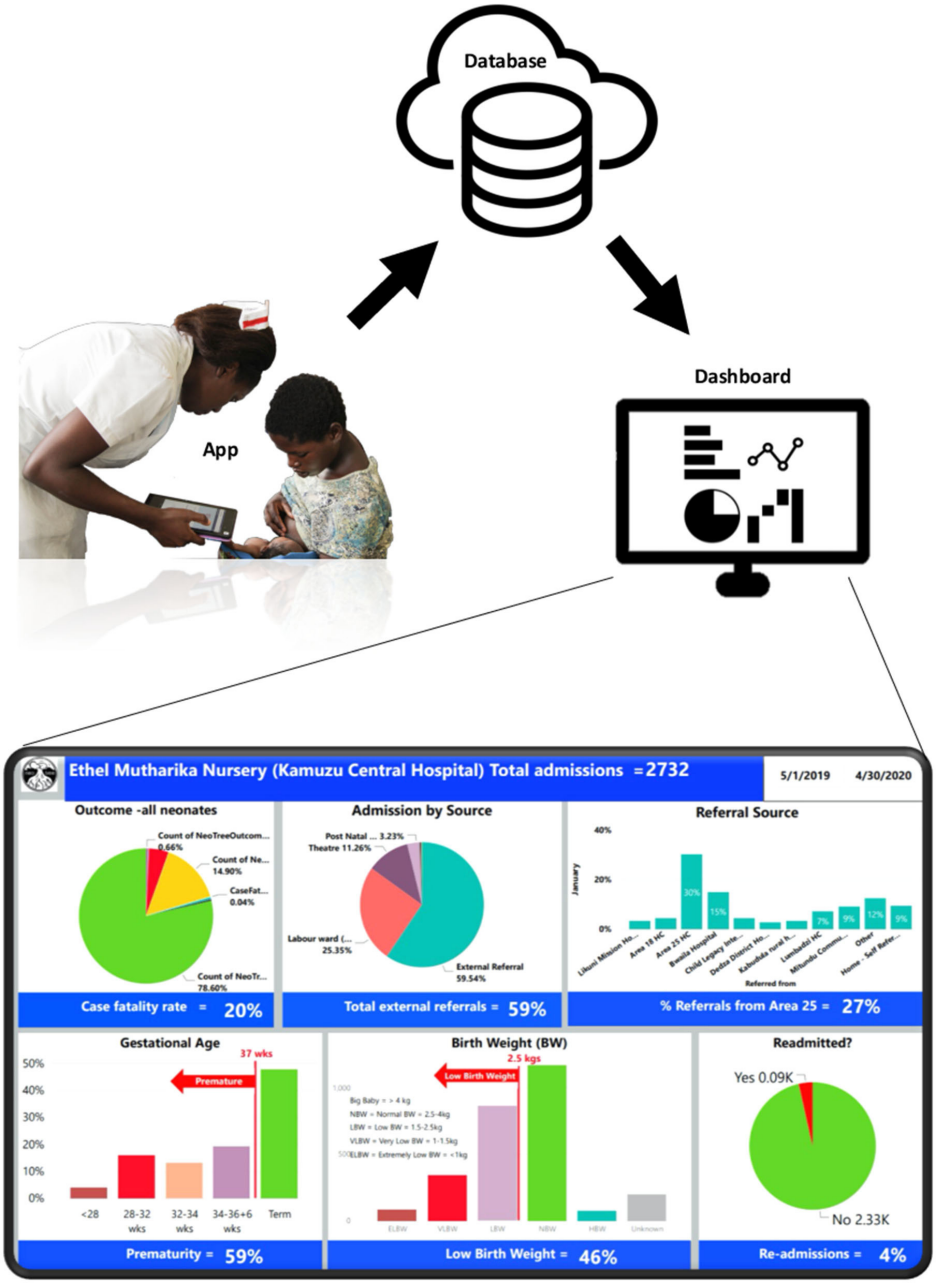
NeoTree system components and dashboard summary screens displayed on the neonatal unit.

The NeoTree system (Figure 2) has been and continues to be developed collaboratively with health workers in Bangladesh (2015), Malawi (19) (2016–2017 and 2019 onwards), and Zimbabwe (23) (2018 onwards) and has been shown to have good acceptability, feasibility, usability (19), and impact on measure of quality of care (22, 23). Data collected on the app are printed in hard copy (including identifiable data) and filed in each patient's paper record. Identifiable data are password protected on the tablet devices before viewing and printing of forms. They are then exported (pseudo-anonymised without identifiable information) *via* a Wi-Fi network to a password-protected cloud database (Malawi only) where they are backed up, de-duplicated and cleaned before matching the admissions to their respective discharges using the NeoTree unique identification number. From there, data are transferred to a Microsoft Power BI dashboard where they are visualised on both a ward screen display (Figure 2) and an online M&M slide deck. Both aspects of the dashboard are refreshable with near to real-time data as often as data are exported from the tablet devices (on average, one time a week). Extraction of data *via* the data dashboard was also password protected, only accessible to account holders. The author (CC) developed the dashboard iteratively with the KCH paediatric department during the first 6 months of this study timeline (25), accessing and providing data for initial M&M meetings, supported by a data analyst (FS) and the NeoTree technical team. The author (YM) also had access to the power BI data dashboard by month 7, where she could drill down through the data and download monthly reports. The author (DN) accessed and supervised data reporting from month 9, January 2020 on the timeline.

### Implementation of the Electronic Data Collection Tool

All permanent nursing staff at the nursery were trained to use the app and issued with user manuals in the week preceding the start of the data collection. Three NeoTree Ambassadors (a paediatric medical officer, a clinical officer, and a senior nurse) were trained to oversee implementation, provide technical support, replenish consumables (paper, toner, and network data), and provide ongoing training for temporary staff or students. Monthly M&M meetings occurred throughout the year of data collection using the dashboard slide deck, which was iteratively improved each month. The online power BI dashboard enabled prospective surveillance of monthly data from months 2–12 including the number of matched outcomes. A large electronic ward screen showing the dashboard was implemented by month 5, and the chief NeoTree Ambassador and deputy head of paediatrics (YM) was issued a Power BI account by month 7. After 1 year, the first 12 months of data were aggregated and visualised on the NeoTree dashboard (Figure 1).

### The Usual Paper Process of Data Collection

Neonatal data, such as age, sex, gestation, place of residence, HIV exposure, place of birth, mode of delivery, etc., are usually recorded manually in the ward register book (Appendix B) by a data clerk from the patients' paper files. This usual process continued throughout this electronic audit, with the clerk assigning each patient in the register; a number which was also written on the patient's file. The clerk then tallied the data columns at the end of each page of the ward register, manually counting the total for each data column per month and entering these totals into aggregate forms (Appendix C). As per usual practise, these aggregate forms were submitted to the data management department within KCH who entered the data into the national information system. Comparisons of NeoTree monthly totals with the data-clerk's monthly totals were conducted retrospectively from month 5.

### Participants

Inclusion criteria for the audit were all neonates admitted within the study period from 1 May 2019 to 30th April 2020. Admission criteria for Ethel Mutharika Neonatal Unit include all medical and surgical patients and neonates with congenital anomalies, all sick neonates, and all neonates <1,800 g or <37-completed-week gestation. Neonates with sepsis, hypoglycaemia, jaundice, and low Apgar score of <7/10 at 5 min are also admitted. Neonates who are well with no signs of infections, feeding well, and with weight above 1,800 g are discharged from the unit.

### Data Analysis

Data were reviewed, filtered, and visualised graphically in a Microsoft Power BI dashboard. Data cleaning and descriptive statistics were executed using R (34) using total NeoTree admissions as the denominator for the analysis of admission data and total NeoTree outcomes as the denominator for outcome data. Results were presented as frequencies (n) and percentages (%) in tables and graphs. Chi^2^ test was used for univariate analyses. For coverage, the proportion of admissions and outcomes captured electronically by NeoTree was calculated by dividing NeoTree totals by the data-clerks totals.

### Ethical Considerations

The study has ethical approval from the University of Malawi College of Medicine Research and Ethics Committee (P.02/19/2613) and the University College London Research Ethics committee (Ref. 6681/001). Permission to compare data with data collected by the ward clerk was sought from the Malawi PI Dr Chiume and the Health Management Information System department at KCH.

## Results

A total of 2,732 patients were admitted to the Neonatal unit electronically on the NeoTree app during the study period. Of these, 2,413 (88.3%) had electronic outcome data recorded through the NeoTree. Data completeness was 100% for all mandatory fields in both admission and outcome forms.

### Admission Data

**Figure 3:**
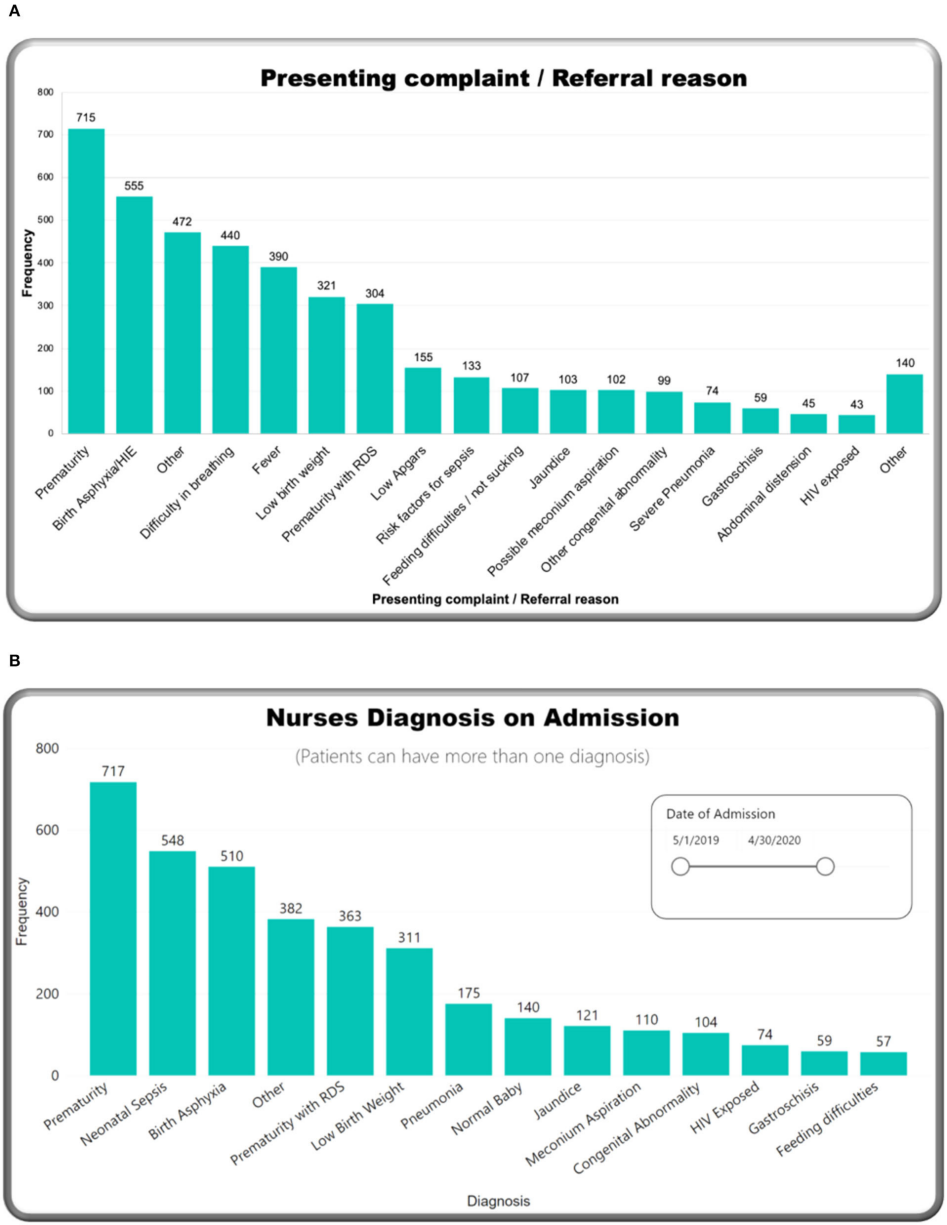
M&M dashboard visuals: **(A)** Presenting complaint/referral reason, **(B)** nursing diagnosis at admission.

**Figure 4:**
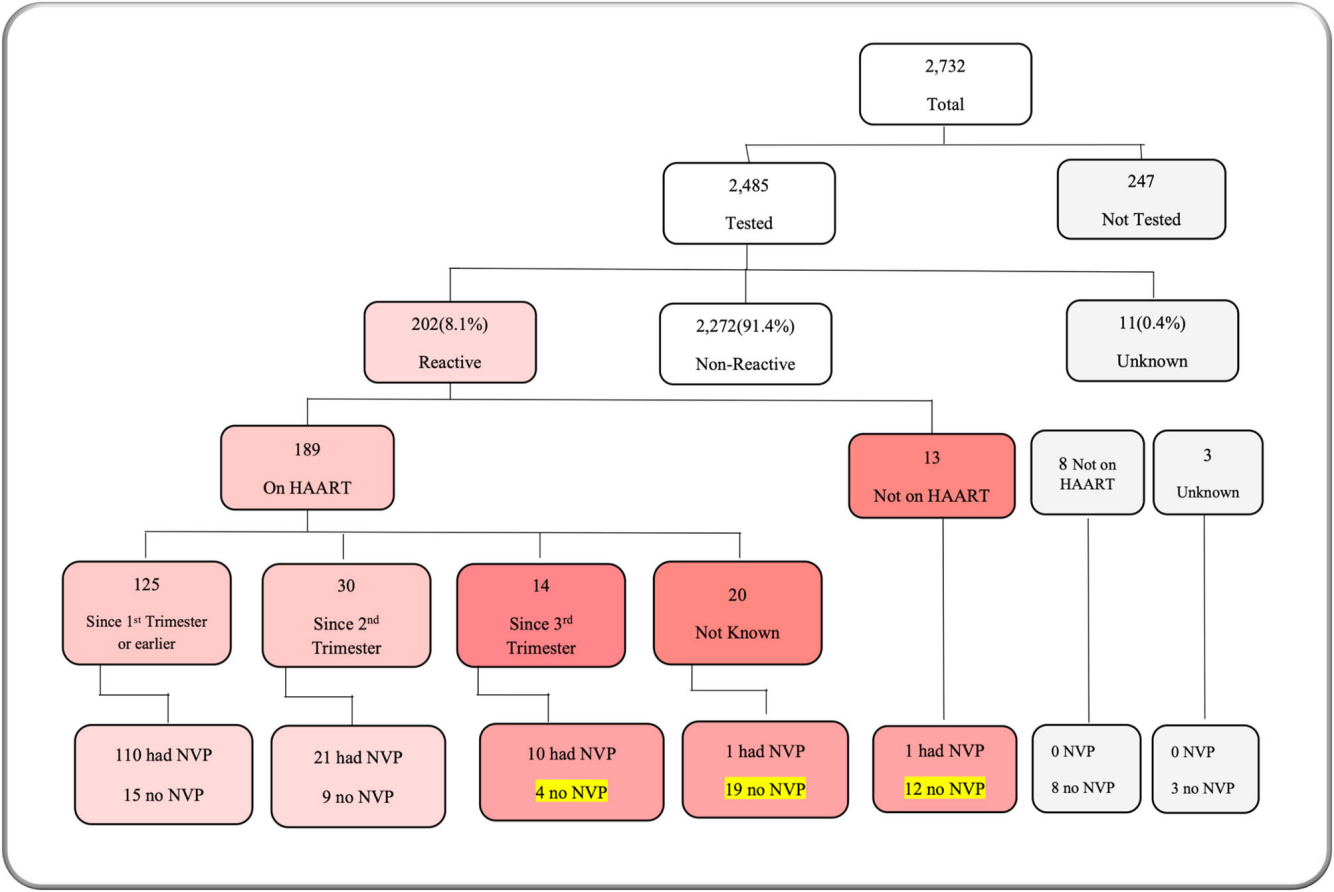
M&M dashboard visual: maternal HIV status. HAART, human anti-retroviral therapy; NVP, nevirapine.

Table 1 describes admission characteristics and vital signs. More than half of the admitted babies were male (55.7%), and born preterm, before 37-week gestational age (53.9%). Approximately two-thirds (67.6%) were <48 h of age at the time of admission. The majority of neonates (60.1%) were admitted following referral from other facilities. Birth weight and admission weight distributions were both negatively skewed. Mean birth weight was 2,392 g [standard de*via*tion (SD) 886 g], and median 1,622 g [inter quartile range (IQR) 2,400–3,100 g]. Mean admission weight was 2,341 g (SD 888 g) with a median of 1,600 g (IQR 2,300–3,000 g). More than half of babies (52.9%) were hypothermic on admission, with a higher proportion of inborn babies admitted hypothermic (60.4%). Prematurity was the most frequent reason for admission (26.2%), followed by neonatal encephalopathy (20.3%) (Figure 3A). Prematurity was also the most common provisional diagnosis made by nurses after the admission assessment (26.2%), followed by neonatal sepsis (20.1%), and then neonatal encephalopathy (18.7%) (Figure 3B). Regarding maternal human immunodeficiency virus (HIV) status, 91.0% of mothers had been tested for HIV before delivery, and 8.1% of those tested had a positive result with 70.8% of HIV-exposed neonates recorded as receiving nevirapine prophylaxis after birth (Figure 4). Of the 59 exposed babies who did not receive nevirapine, 35 were particularly vulnerable to vertical transmission with late, unknown, or no maternal antiretroviral therapy (highlighted in Figure 4). In addition, maternal syphilis status, 78.4% mothers were tested for syphilis before delivery, and of these 2.7% had a positive test. Out of those who were tested positive, only half (50.9%) received syphilis treatment.

**Table 1:** Patient characteristics and vital signs of admissions.

**Characteristics**	**Findings**	***n***	**Missing *n* (%)**	**Complete (%)**
**Demographics**				
**Gender**, ***n*** **(%)**		2,732	0	100
Female	1,208 (44.2)			
Male	1,521 (55.7)			
Not sure (ambiguous genitalia)	3 (0.1)			
**Age**, ***n*** **(%)**		2,215	517 (19)	81
<48 h	1,848 (67.6)			
>48 h	367 (13.4)			
**Type of birth**, ***n*** **(%)**		2,732	0	100
Singletons	2,322 (85.0)			
Twins	389 (14.2)			
Triplets	21 (0.8)			
**Admitted from**, ***n*** **(%)**		2,732	0	100
Within Kamuzu Central Hospital	1,090 (39.9)			
Outside Kamuzu Central Hospital	1,642 (60.1)			
**Weight and gestation**				
**Birth weight (g)**, ***n*** **(%)**		2,535	197 (7.2)	92.8
Mean (SD)	2,392 (886.2)			
Median (IQR)	1,622 (2,400–3,100)			
Range	500–5,500			
<2,500 g [*n* (%)] (Low BW)	1,285 (50.6)			
>2,500 g [*n* (%)] (Normal BW)	1,250 (49.4)			
**Admission weight (g)**, ***n*** **(%)**		2,732	0	100
Mean (SD)	2,341 (888.3)			
Median (IQR)	1,600 (2,300–3,000)			
Range	505–5,800			
<2,500 g [*n* (%)]	1,513 (55.4)			
≥2,500 g [*n* (%)]	1,219 (44.6)			
**Gestation (weeks)**		2,731	1 (0.1)	99.9
Mean (SD)	34.97 (4.0)			
Median (IQR)	32 (31–33)			
Range	22–42			
<28 weeks [*n* (%)]	108 (4.0)			
<37 weeks [*n* (%)]	1,424 (52.1)			
Term (≥37 weeks) [*n* (%)]	1,307 (47.9)			
**Vital signs on admission, mean (SD)**				
Heart rate (beats/minute)	137.7 (24.7)	2,728	4 (0.1%)	99.9%
Respiratory rate (breaths/minute)	55.42 (18.8)	2,732	0 (%)	100%
Oxygen saturations in air (%)	87.5 (15.6)	2,728	4 (0.1%)	99.9%
Temperature (°C)	36.4 (1.4)	2,729	3 (0.1%)	99.9%
Blood sugar (mmol/l)	120.2 (71.1)	2,486	246 (9.0%)	91.0%
**Abnormal vital signs on admission**, ***n*** **(%)**
Tachycardic (heart rate >160 beats/min)	389 (14.3%)	2,728	4 (0.1%)	99.9%
Hypoxic (oxygen saturations <90% in air)	870 (31.9%)	2,728	4 (0.1%)	99.9%
Tachypneic (respiratory rate >60 breaths/min)	787 (28.8%)	2,732	0(%)	100%
Hypothermic (temperature <36.5°C)	1,277 (52.9)	2,732	0	100
Hypothermic–Inborn	659 (60.4)	1,090		
Hypothermic–Out-born	618 (37.6)	1,642		
Hyperthermic (temperature >37.5°C)	2,123 (77.8%)	2,729	3 (0.1%)	99.9%
Hypoglycaemia (blood sugar <2.6 mml/l)	45 (1.8%)	2,486	246 (9.0%)	91.0%

*BW, birth weight; IQR, interquartile range; LBW, low birth weight; LMP, last menstrual period; RDS, respiratory distress syndrome; SD, standard deviation*.

### Outcome Data

**Figure 5:**
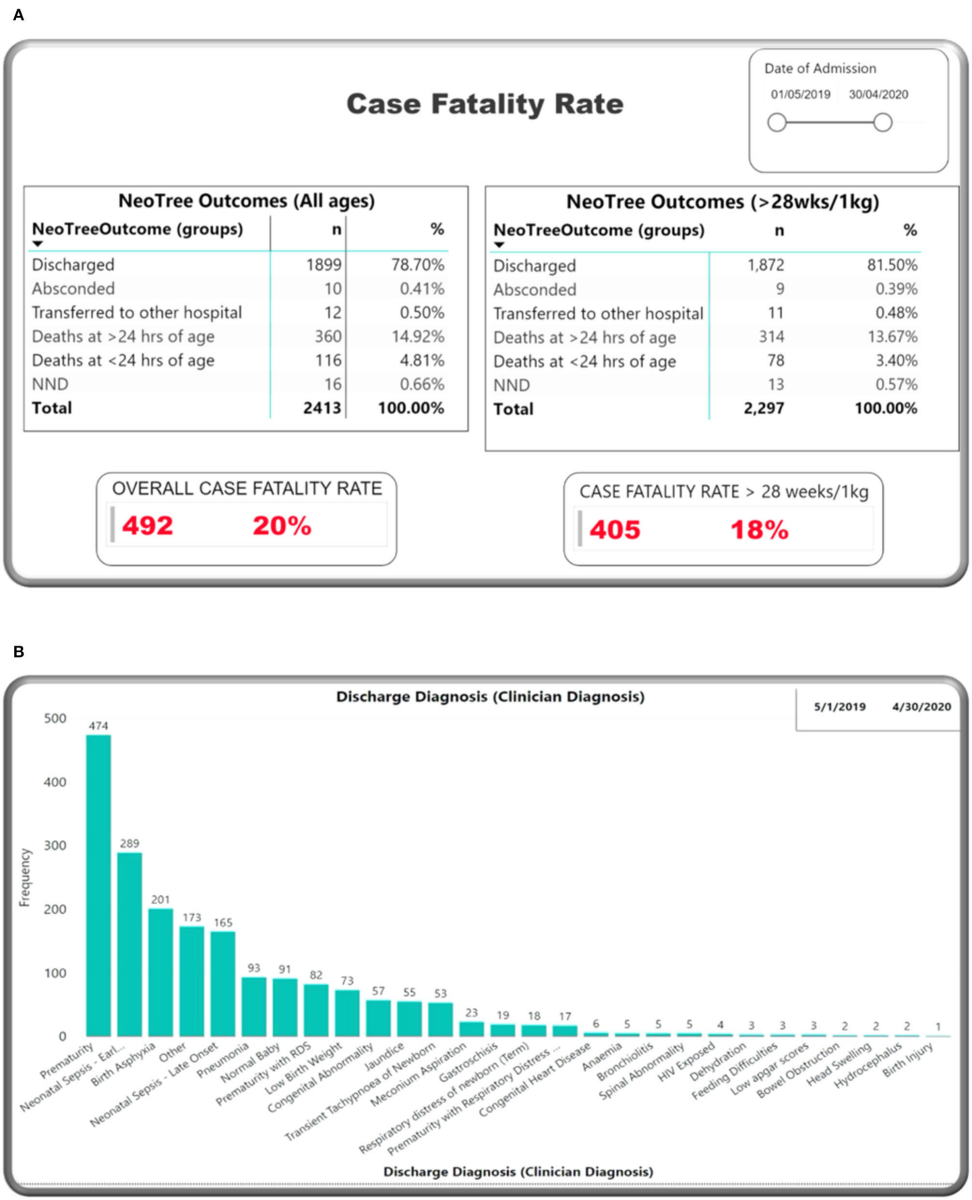
M&M dashboard visuals: **(A)** Outcomes, **(B)** discharge diagnosis.

Figure 5A shows neonatal outcomes during the study period on the dashboard. Using total outcomes as the denominator, 80.0% neonates survived, including 78.7% that were discharged, 0.5% referred to another hospital, and 0.4% that left against medical advice (absconded). There were 492 deaths in total (20.4%), giving a total case fatality rate (CFR) of 204 per 1,000 admitted newborns for the study year. Initially, deaths were reported as “NNDs” (neonatal deaths), but, early in the study (05 May 2019), the outcome field of the app was changed to allow the more specific selection of “death at <24 h” or “death at more than 24 h,” hence total deaths for the year include all these groups. If we exclude neonates that were <28 weeks or 1,000 g in weight (and, therefore, typically very unlikely to survive in settings such as this), and then the CFR drops to 168 per 1,000 babies admitted. There were no babies “brought in dead” or stillbirths reported in this audit. Stillbirths are recorded under Obstetrics data and not under neonatal.

**Figure 6:**
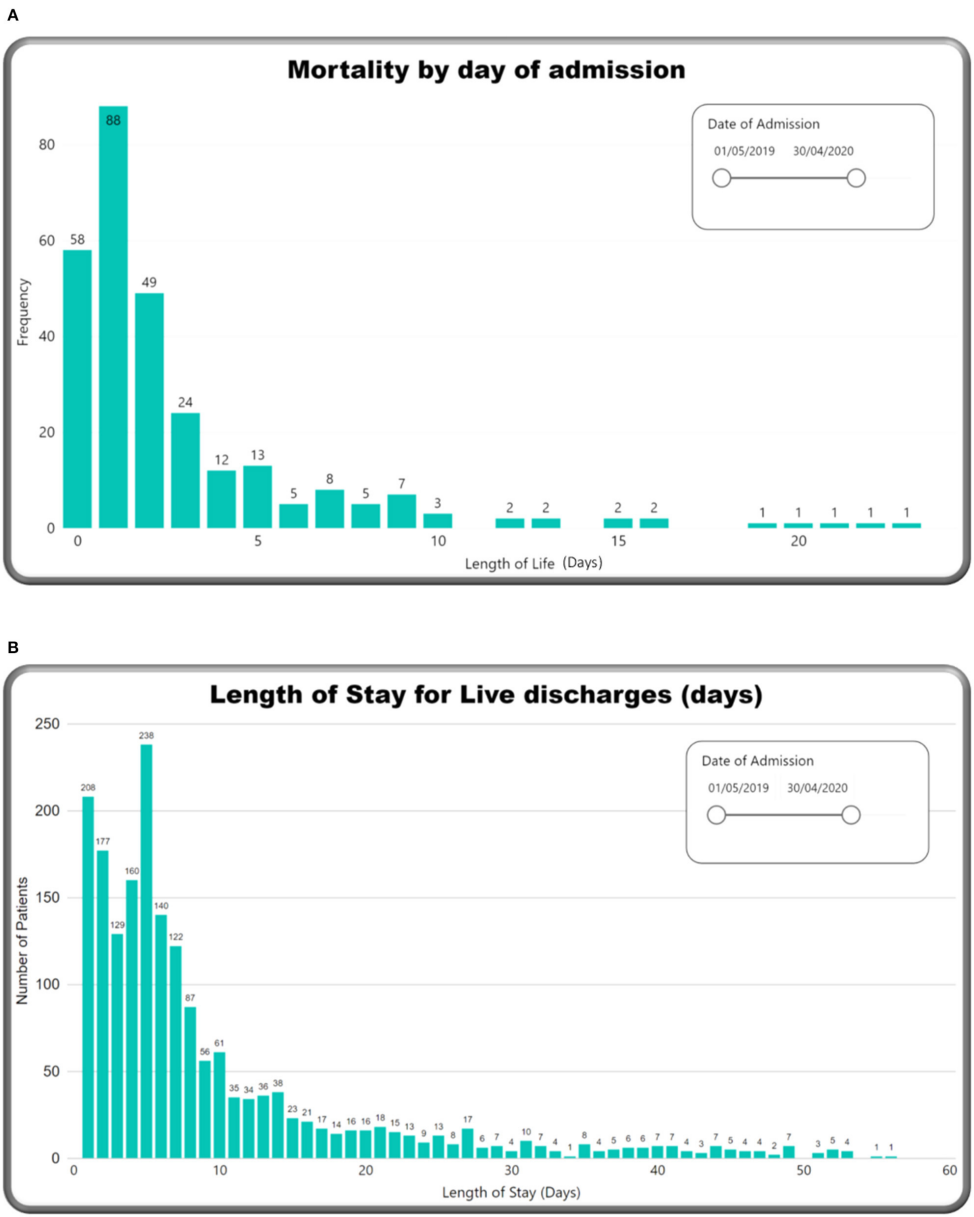
M&M dashboard visuals: **(A)** Mortality by day of admission, **(B)** length of stay.

Figure 5B summarises clinician diagnoses at discharge for all surviving neonates. The top three diagnoses were prematurity (19.6%), neonatal sepsis (12.0%), and neonatal encephalopathy (8.3%). Of note, each neonate can have more than one diagnosis. Almost 50% of surviving neonates were discharged by Day 6 (Figure 6B).

Table 2 outlines the causes of death at the KCH neonatal unit, with corresponding CFRs. The commonest causes of death were prematurity with RDS (39.4%), neonatal sepsis (23.6%), and neonatal encephalopathy (16.2%). Almost 40% of all neonatal deaths occurred within 72 h of being admitted (Figure 6A). The highest CFRs per 1,000 cases were prematurity with RDS (616 per 1,000), followed by congenital abnormality (270 per 1,000), neonatal sepsis (238 per 1,000), and neonatal encephalopathy (179 per 1,000).

**Table 2:** Cause of death and case fatality rates.

**Cause of death^a^**	**Total neonates with diagnosis^b^, *n* (%)**	**Percentage deaths from total cases^b^, *n* (%)**	**Case fatality rate per 1,000 cases**	**% deaths from total deaths (*n* = 492), *n* (%)**
Prematurity	1,241 (51.4)	58 (4.7)	47	58 (11.7)
Prematurity with RDS	315 (13.1)	194 (61.6.)	616	194 (39.4)
Neonatal encephalopathy	448 (18.6)	80 (17.9)	179	80 (16.2)
Neonatal sepsis	488 (20.2)	116 (23.8)	238	116 (23.6)
Congenital anomaly	89 (3.6)	24 (27.0)	270	24 (4.8)
Total	2,413 (100.0)	492 (20.4)	204	492 (100.0)

a *Causes of death are not mutually exclusive, i.e., each neonate can have more than one cause of death*.

b *Diagnosis totals were nurses diagnoses on admission for all neonates with an outcome, except for prematurity, which was total number of gestation of <37 weeks for which outcomes were available*.

Table 3 shows CFRs by patient characteristics other than primary diagnosis. The lower the admission weight, birth weight, and gestational age, the higher the CFR. The admission weight groups 1,000–1,500 g, and 1,500–2,500 had the most deaths, contributing to almost one third of deaths each (31 and 29%). The birth weight groups 1,000–1,500 and 1,500–2,500 g had the most deaths; (28 and 26% of deaths, respectively), and more deaths occurred in gestation of 28–32 weeks (30%) and term babies (28%). Statistically significant factors associated with higher CFR (Table 4) were out born (*p* = 0.039), admission weight <2,500 g (*p* < 0.001), low birth weight (*p* < 0.0001), prematurity (*p* < 0.0001), and hypothermia (*p* < 0.0001). Out-born hypothermic infants had significantly higher CFRs than inborn hypothermic infants (*p* < 0.0001).

**Table 3:** CFRs in different patient groups.

**Patient group**	**Total neonates *n* (%)**	**Group percentage deaths, *n* (%)**	**CFR/1,000 patients in group**	**Group % deaths from total deaths (*n* = 92), *n* (%)**
**Admission weight group**				
<1,000 g^a^	104 (4)	74 (71.1)	711	74 (15.0)
1,000–1,500 g^b^	352 (15)	154 (43.8)	438	154 (31.3)
1,500–2,500 g^c^	865 (31)	143 (16.5)	165	143 (29.0)
2,500–4,000 g^d^	1,010 (42)	117 (11.6)	116	117 (23.7)
>4,000 g^e^	82 (3)	4 (4.9)	49	4 (0.8)
Total	2,413 (100)	492 (20.4)	204	492 (100)
**Gestational age group**				
<28 weeks	94 (4)	71 (75.5)	755	71 (14.4)
28–32 weeks	373 (15)	146 (38.9)	389	146 (29.7)
32–34 weeks	315 (13)	56 (17.8)	178	56 (11.4)
34–36+6 weeks	458 (19)	80 (17.5)	175	80 (16.2)
Term (≥37 weeks)	1,172 (49)	139 (11.9)	119	139 (28.3)
N/A	1 (0.04)	0 (0)	0	0 (0)
Total	2,413 (100)	492 (20.4)	204	492 (100)
**Referral centre**				
Area 25 health centre	432 (30.0)	78 (18.1)	181	78 (24.2)
Bwaila hospital	215 (14.9)	33 (15.3)	153	33 (10.2)
Mitundu community hospital	130 (9.0)	44 (33.8)	338	44 (13.7)
Lumbadzi HC	100 (6.9)	15 (15.0)	150	15 (4.7)
Other referral centres	565 (39.2)	152 (26.9)	269	152 (47.2)
Total	1,442 (59.8)	322 (22.3)	223	322 (65.4)

a *Extremely low birth weight*.

b *Very low birth weight*.

c *Low birth weight*.

d *Normal birth weight*.

e *High birth weight*.

**Table 4:** Univariate comparisons (Chi^2^ analysis).

**Patient group**	**Total neonates, *n* (%)**	**Percentage deaths within individual groups, *n* (%)**	**CFR/1,000 patients in group**	**% deaths from total deaths (*n* = 492), *n* (%)**	
**Admission source**					
Inborn	971(40.2)	170 (17.5)	175	170 (34.6)	*P* = 0.0046
Out born	1,442 (59.8)	322 (22.3)	223	322 (65.4)	
Total	2,413 (100)	492 (20.4)	204	492 (100)	
**Admission weight (AW)**					
AW <2,500 g	1,321 (55)	371 (28.1)	281	371 (75.4)	*P* < 0.00001
AW ≥2,500 g	1,092 (45)	121 (11.1)	111	121 (24.6)	
Total	2,413 (100)	492 (20.4)	204	492 (100)	
**Birth weight < /> 2.5**					
LBW <2.5	1,121 (46.5)	324 (28.9)	289	324 (65.9)	*P* < 0.00001
NBW >2.5	1,121 (46.5)	123 (11.0)	110	123 (25.0)	
Total	2,242 (92.9)	447 (20.0)	200	447 (90.9)	
**Gestation <37 vs**. **>36**					
Prem (<37 weeks)	1,240 (51)	353 (28.5)	285	353 (71.7)	*P* < 0.00001
Term (≥37 weeks)	1,172 (49)	139 (11.9)	119	139 (28.3)	
N/A	1 (0.04)	0 (0)	0	0 (0)	
Total	2,413 (100)	492 (20.4)	204	492 (100)	
**Admission temperature**					
Hypothermic	1,116 (46.2)	321 (28.8)	288	321 (65.2)	*P* < 0.00001
Normothermic (inch. Hyper)	842 (34.9)	96 (11.4)	114	96 (19.5)	
Total	1,958 (81.1)	417 (21.3)	213	417 (84.6)	
**Hypothermic inborn vs. out born**					
Hypothermic inborn	583 (24.2)	138 (23.7)	237	138 (28.0)	*P* = 0.0001
Hypothermic out born	533 (22.1)	183 (34.3)	343	183 (37.2)	
Total	1,116 (46.2)	321 (28.7)	287	321 (65.2)	

**Figure 7:**
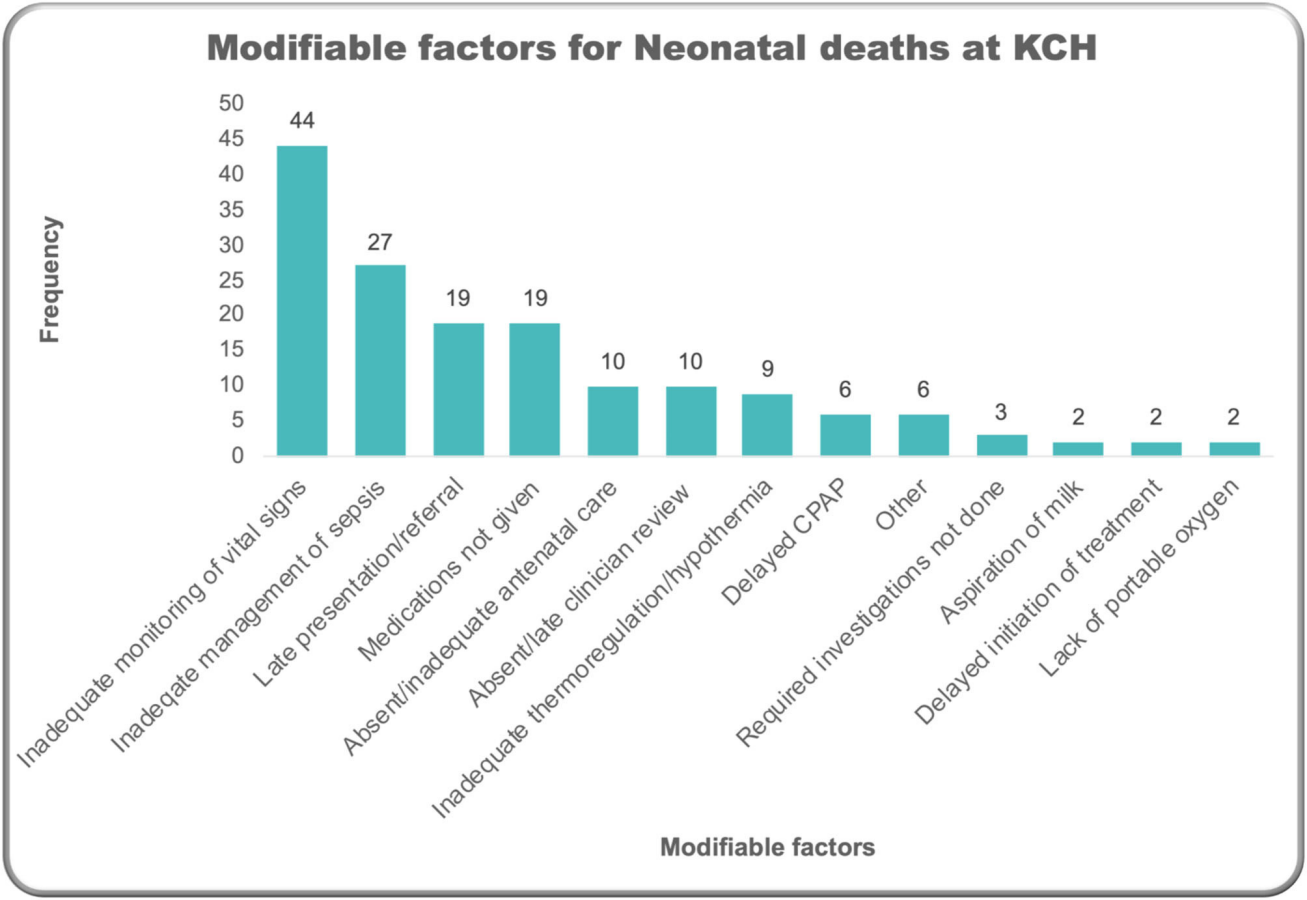
Modifiable factors around deaths.

In around a quarter (26%) of all deaths recorded *via* the NeoTree, healthcare professionals recorded perceived modifiable risk factors for deaths. In other words, factors that were perceived by the healthcare professionals involved in the baby's care and recording the details of the baby's death that both contributed to the baby's death and that they thought were, in some way, preventable. The most frequently recorded perceived modifiable factor contributing to a baby's death was inadequate monitoring of vital signs (9% of all deaths) and suboptimal management of sepsis (5% of deaths) (Figure 7). These included one death where antibiotics were indicated but not prescribed or given, 19 deaths where antibiotics were prescribed but administered late, and 6 deaths where prescribed antibiotics were not given at all.

### Monthly Trends

**Figure 8:**
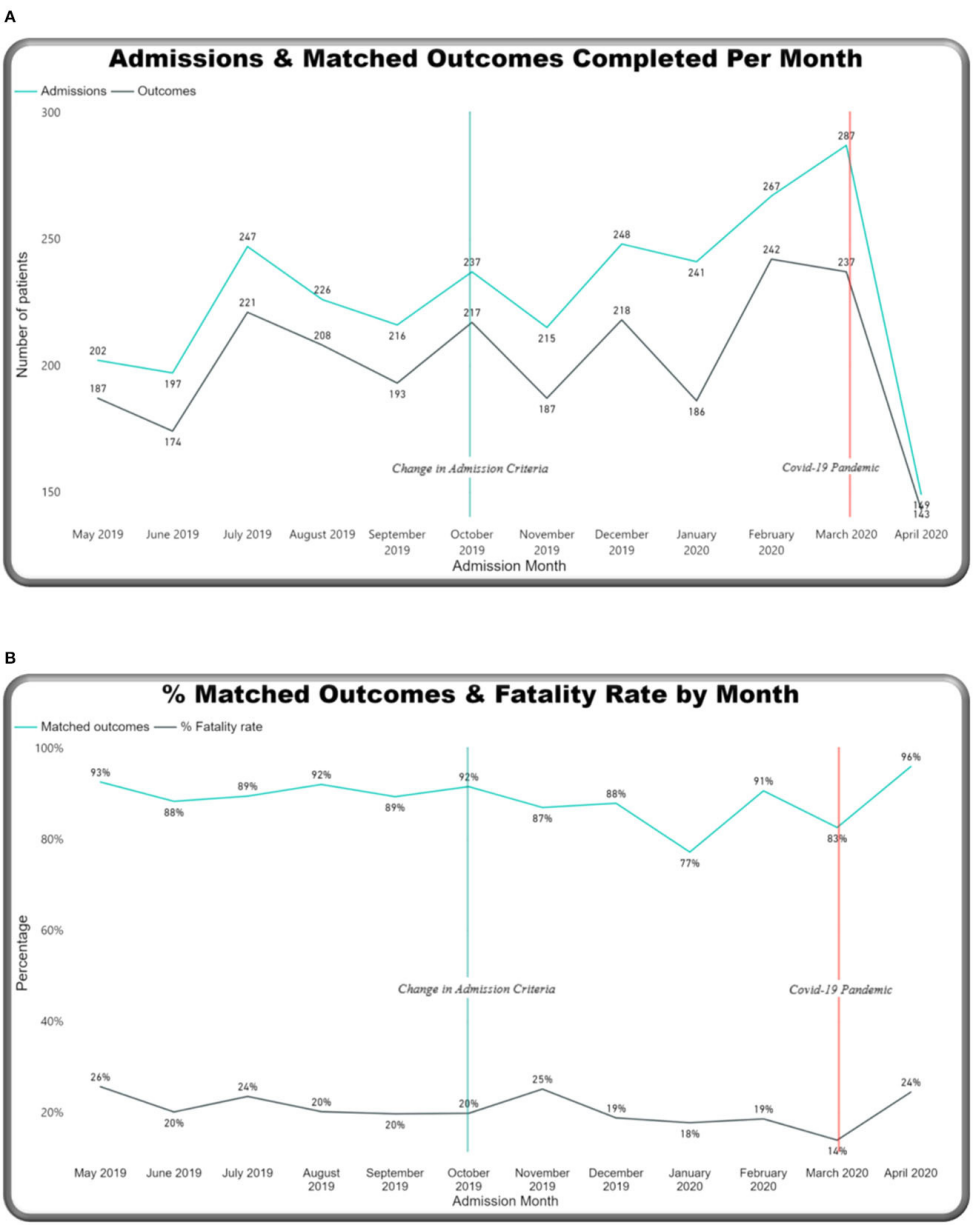
M&M dashboard monthly trends: **(A)** Admissions & matched outcomes, **(B)** % matched outcomes and case fatality rates.

**Figure 9:**
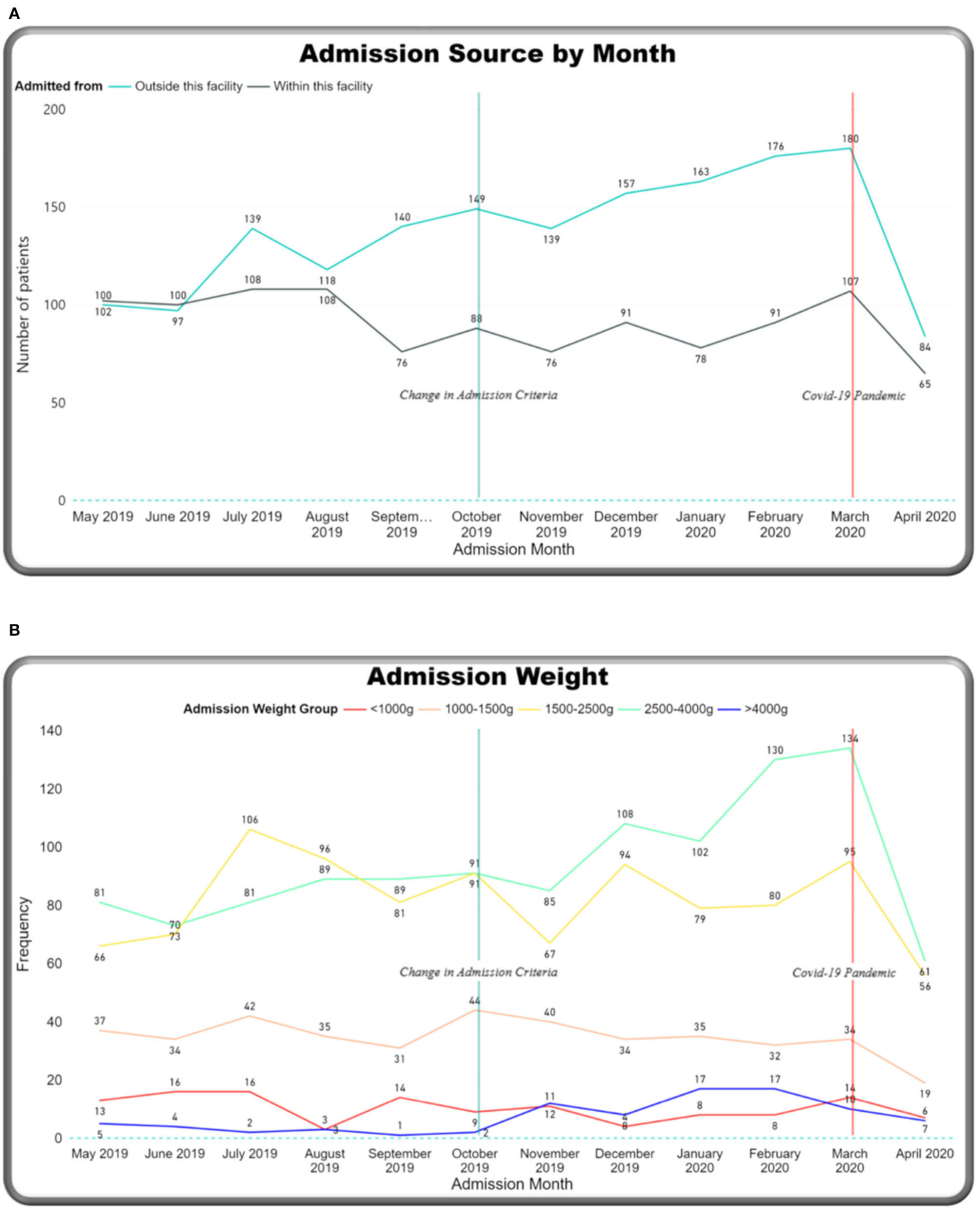
M&M dashboard monthly trends: **(A)** Admission source, **(B)** admission weight.

**Figure 10:**
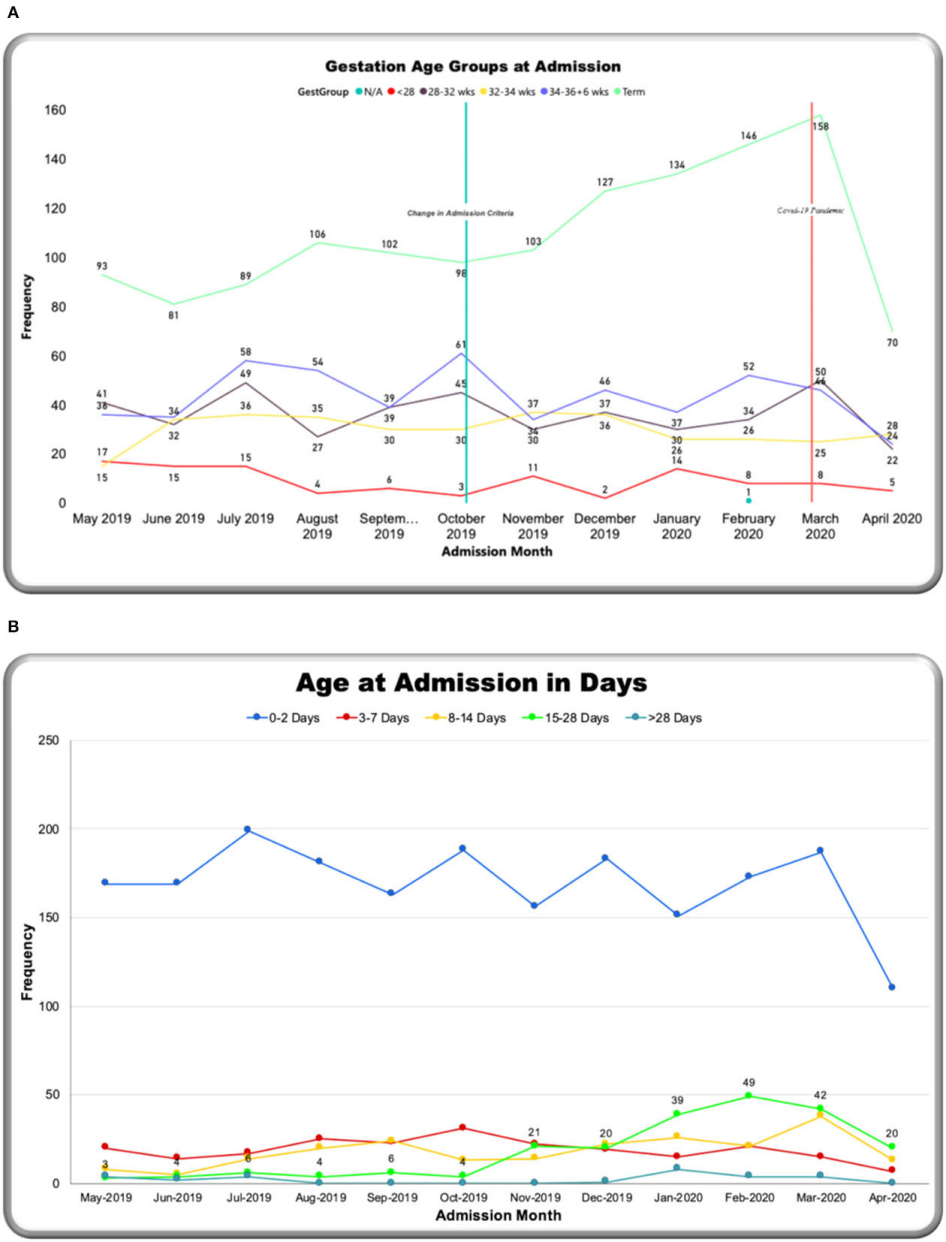
M&M dashboard monthly trends: **(A)** Gestation, **(B)** age at admission in days.

**Figure 11:**
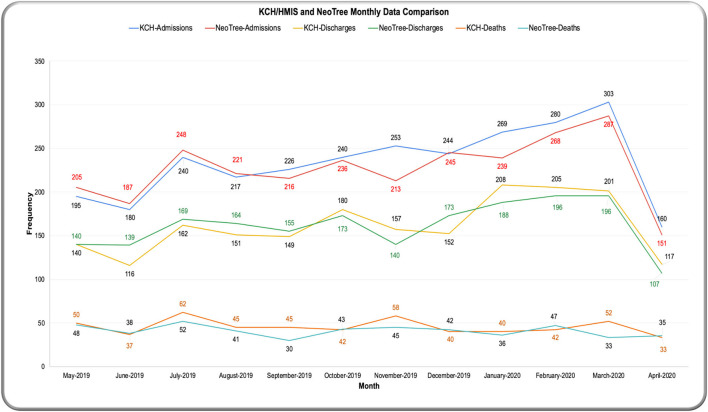
Coverage—admissions and outcomes collected by KCH data clerk and NeoTree.

Monthly trends were tracked on the dashboard relating to two main events: an increase in admission criteria age (from 14 days up to 28 days) in October 2019 and the start of the COVID-19 pandemic in March 2020 (Figures 8–11). Over the year, the number of admissions to the neonatal unit each month ranged from 149 to 287 per month (mean: 228) (Figure 8A). On average, 26.6 outcomes were not completed each month. Admission numbers increased after the change in admission criteria and then reduced significantly in the month of April 2020, after the first cases of COVID 19 were reported in Malawi (see Figure 1). Figure 8B shows percentage-matched outcomes over the year, which ranged from 77 to 96%, and the monthly CFR ranged from 140 per 1,000 to 26 per 1,000 with the highest CFR recorded in May 2019 (260 per 1,000). Lower rates of matched outcomes appear to occur with lower CFRs. Figure 9A shows increasing outside referrals compared to inborn admissions after the expansion of the admission age criteria in October, with a decline in both out-born and inborn patients in the month of April 2020 (with a more pronounced decline in out-born referrals). There was an expected concomitant increase in admissions, weighing 2,500–4,000 g (Figure 9B), of term gestation (Figure 10A), and patients aged 15–28 days (Figure 10B) after the change in admission criteria.

### Coverage

Overall, NeoTree captured 2,732/2,807 (97%) admissions, 1921/1938 (99%) discharges and 498/546 (91%) deaths recorded by the ward clerk (where “discharges” include transfers out and absconders). Looking at monthly coverage over the year (Figure 11), the NeoTree captured more data than the data clerk in the first quarter, each month capturing, on average, 13 more admissions in the first 4 months and 12 more discharges in the first 5 months. Most of the year (7/12 months), the ward clerk recorded more deaths than the NeoTree, on average six more deaths than NeoTree per month. Out of 2,484 total outcomes collected by the ward clerk, 546 were deaths, giving a paper-based CFR of 220 per 1,000 for all admissions that had an outcome recorded by the ward clerk.

## Discussions

This paper describes the collection of crucial newborn data by healthcare professionals at the point of care *via* an electronic app NeoTree. We report a digital CFR of 204 per 1,000 at KCH with prematurity with RDS, neonatal sepsis, and neonatal encephalopathy being the commonest causes of death. Prematurity with RDS had the highest CFR. Key perceived modifiable factors associated with neonatal deaths were inadequate monitoring of vital signs and suboptimal management of neonatal sepsis. Whilst most women were tested antenatally for HIV, fewer were tested for syphilis, and only 70% of HIV-exposed infants were documented to have received nevirapine prophylaxis. The dashboard allowed prospective tracking of monthly trends in relation to a change in admission criteria and a global pandemic. Coverage of admissions and discharges was high, suggesting representativeness of the audit; however, 9% of deaths were not captured, suggesting a possible underestimation of CFR and a need to revise data collection processes around neonatal deaths.

### Admission Data

Differences between “presenting complaint/referral reason” entered at the beginning of the app and “nurses admission diagnoses” entered at the end of the app are expected and likely to reflect the thorough clinical assessment by the admitting nurse *via* NeoTree. Of note, the number of neonates presenting with encephalopathy (555) dropped to 510 after their admission assessment by a nurse, and only 210 were diagnosed with this later by a clinician at discharge. This may be due to the baby becoming more active after admission, with neurological signs of encephalopathy improving, but also suggests there may be scope for training in neurological examination of infants. Although instructions and images on how to perform Thompson Score (TS) were configured in the app during the study and 334 scores were captured, formal training on how to perform this score at KCH is planned.

The proportion of HIV-exposed neonates (8.1%) is in keeping with high national prevalence of HIV at 9.2% (2). Our data indicate that almost one third of admitted HIV-exposed neonates had not received HIV prophylaxis (nevirapine) by the point of admission, increasing their likelihood of vertical infection. This might be because HIV-positive mothers in Malawi are usually given a bottle of nevirapine antenatally, and it is their responsibility to administer it to their babies straight after birth. However, this might be difficult for mothers who are moribund post theatre, or guardians may not have been informed this was required. Since this audit, a reminder to administer nevirapine (if not done already) was configured into the admission form, but a review of “prevention of mother-to-child transmission” at KCH may also be indicated, perhaps conferring nevirapine administration to midwives when mothers are recovering. Collecting nevirapine coverage at discharge *via* NeoTree might also be helpful; however, this would not capture the timeliness of NVP administration, which is important for its effectiveness.

Whilst 91% of mothers were tested for HIV, only 78% were tested for syphilis antenatally. Although this is an improvement from 73% in the Zomba pilot (19), the opportunity to potentially prevent neonatal syphilis was missed in one fifth of mothers. This emphasises an enduring gap between antenatal HIV and syphilis screening across Africa due to lack of awareness, funding, and political will to address antenatal syphilis (35). Our findings give weight to a call for increasing availability of maternal syphilis rapid diagnostic test. Leveraging the NeoTree app's diagnostic algorithm to identify and treat newborns of unscreened mothers is also recommended.

### Outcome Data

The CFR produced by NeoTree for all neonatal admissions to KCH, in its first year, was 204 per 1,000. This is lower than the CFR of 220 per 1,000 calculated from the ward logbook during the same period and a recent manual/paper audit in the same hospital, which reported a CFR of 230 per 1,000 (20). This is likely related to 12% of the NeoTree admissions in this study, lacking an electronic outcome thus underestimating the number of deaths. It could also be due to the data clerk omitting some admissions that go on to survive. Coverage data in this study suggest that 9% of deaths recorded by the data clerk were missed by NeoTree, suggesting that the missing outcomes are more likely to be deaths rather than live discharges. Future NeoTree usability studies could, therefore, explore factors contributing to why NeoTree forms are less likely to be completed on babies who have died to improve the reliability of NeoTree's CFR. In the first quarter of the audit period, NeoTree captured more admissions and discharges than the clerk, perhaps representing a honeymoon period for the intervention or that files were being removed from the ward for ongoing research studies before they could be logged by the clerk. Longitudinal monitoring of coverage past this initial first year is recommended to assess whether engagement can be sustained once NeoTree is embedded.

Irrespective of possible underestimation, our digital CFR of 204 per 1,000 still exceeds that of neonatal units in other Sub-Saharan African countries such as South Sudan (135 per 1,000) (36), Ghana (202 per 1,000) (37), Rwanda (133 per 1,000) (38), and South Africa (140 per 1,000) (31). This difference may be explained by these countries only reporting deaths for babies over 28 weeks and/or 1,000 g (> 1,250 g in the Sudan study). Indeed, when we restrict our data to these parameters, our CFR reduces to 168 per1,000. Malawi's health system was ranked poorly at 185th by the WHO health system ranking 2020 (32), lower than these aforementioned countries (134th, 135th, 172nd, and 175th, respectively), hence, their lower CFRs might be due to better overall quality of their health systems. Indeed, neonatal CFR for a unit in Nigeria was higher than KCH at 370 per 1,000 (32), consistent with Nigeria's health system ranking lower than Malawi at 187th (31). As expected, CFR was significantly higher in neonates with lower gestation, birth weights, admission weights, and admission temperatures, with the latter being the risk factor most amenable to impact. QI to improve admission hypothermia at KCH is already ongoing, with nurses using a low-cost transport incubator to reduce neonatal heat loss between a labour ward and a neonatal unit. Our findings that significantly more deaths occurred in out-born babies is consistent with the previous manual KCH audit (21), and our finding that significantly more out-born hypothermic babies die than inborn hypothermic babies suggests that in-transit sources of hypothermia between facilities might also be a quality improvement target area for reducing deaths.

The top three clinician diagnoses in neonates that survived to discharge were prematurity, early onset neonatal sepsis, and neonatal encephalopathy similar to other African neonatal units (12, 36, 38). The commonest causes of death were prematurity with RDS, neonatal sepsis, and neonatal encephalopathy, which differ from Zomba central hospital (ZCH), where the commonest cause of death was neonatal encephalopathy (58%). This may reflect later presentations to ZCH from a wider more rural catchment area, leading to higher rates of obstructed labour and, therefore, asphyxia. Comparisons of NeoTree causes of death with the previous KCH study (20) are difficult because it reports cause of death as mutually exclusive, despite multiple diagnoses contributing to each death. Accurate reporting of cause of death using clinical judgement alone is problematic (33). An advantage for NeoTree is that causes and modifiable factors around deaths were immediately recorded at the bedside when deaths occurred, reducing likelihood of reporting bias.

Despite providing a specific field option to record babies “brought in dead” (BID) to KCH and later introduction of a specific BID script to streamline only essential data capture for these infants (November 2019), no BIDs were recorded on the NeoTree during the study. This may have been due to the prioritisation of documentation of live admissions, reluctance to include these in daily statistics or a technical error with exporting scripts. The complete absence of any data capture for these deaths occurring between birth and arrival underlines long-standing difficulties in capturing early neonatal deaths in these settings. Understanding modifiable factors around these is crucial to reducing overall NMR and may require more collaboration between maternal and neonatal departments.

Inadequate vital signs monitoring was the most frequently reported modifiable factor around deaths that were captured, suggesting current nurse to patient ratios (as low as 1 nurse to 40 infants) may be insufficient for achieving the target MOH for hourly minimum frequency (39). The real-time nature of this audit prompted two focused vital signs audits (Figure 1), demonstrating the NeoTrees capacity for timely, targeted quality improvement and, in this case, multiple completed audit cycles. Late administration of antibiotics was the most common sepsis management issue, suggesting that timely antibiotic administration might have saved 19 of the 116 sepsis-related deaths. In Zimbabwe, preliminary NeoTree audits of antibiotic administration have shown improved adherence to appropriate first-line antibiotic therapy (22), and laboratory forms were piloted, aiming to reduce blood culture turnaround time and improve timeliness of antibiotics (23). Responding to learning from this pilot (23) deployment of laboratory forms at KCH is now underway and could facilitate timeliness of antibiotic administration according to sensitivities, in conjunction with a baseline antibiotic audit.

The dashboard enabled real-time tracking of monthly data trends around key events, for example, there was an expected drop in both inborn admissions and outside referrals after the COVID-19 pandemic started as “COVID-measures” were put in place. Only 4 weeks of pandemic-associated data are presented here, and a separate time-series analysis due for publication examines data patterns relating to the first cases of COVID19 in more detail (40). Notably, the highest matched outcomes occurred at this point when staff were on strike. This is likely due to the drop in admission numbers and a lower workload, giving even minimal staff more time to enter outcomes electronically. The previous drop in matched outcomes reported in December 2019 could be attributed to the absence of students on the ward during Christmas holidays. Reliance on students for clinical work and complete NeoTree was also seen in the Zomba pilot; hence, findings from both hospitals have highlighted the ongoing staffing deficit in Malawian neonatal units.

### Strengths and Limitations

To our knowledge, this is the first published large prospective point-of-care electronic neonatal audit from a low resource setting, and the only one that reports data using a dashboard. Because these data were collected by health professionals at the point-of-care, it is likely to be of higher quality than previous audits using retrospective data collection *via* a data clerk. Strengths of the study include the novel dashboard reporting method, high data coverage, and completeness of data. This audit includes all hospitalised neonates without excluding congenital abnormalities as in previous studies (20) and is the first audit including real-time reporting of modifiable factors around deaths when they occur. Whilst collecting quality data, the NeoTree, crucially, also provided clinical decision support for nurses as they admitted babies. Eight Morbidity and Mortality (M&M) meetings were done using dashboard data during this study. The dashboard feedback loop allowed monthly trends on the dashboard to be discussed regularly with paediatric and obstetric departments, leading to a range of timely quality improvement initiatives, and, in the case of vital signs monitoring, the completion of two audit cycles. Power outages and subsequent network failures were successfully remedied by the use of an Uninterrupted Power Supply (UPS), which sustained power for the system for more than 30 min during blackouts.

Limitations include that this is a single-centre study, limiting the generalisability of the findings, and lack of outcome data for 12% may limit the reliability of case fatality rates. Gestational ages were largely estimated using maternal fundal height (40%), which is unreliable (41), and overuse of the “other” category may have limited the reliability of diagnoses. The latter issue could be a focus for future user testing or training or handled by queries in the data pipeline. Modifiable factors around deaths were only the perceptions of the nurses completing NeoTree death forms, so they could have been subjected to bias, but real-time capture of these electronically when deaths occur increases data quality and reduces reporting bias. Standardised reporting and classification of modifiable factors is now recommended by the updated Malawian neonatal guidelines (8), hence replacing the current free-text field, with a dropdown of these codes is recommended. Only one incident of theft of the mobile tablet devices occurred during this study, and the situation was addressed by the local team, deciding a clear set of rules and regulations for storing and keeping equipment safe in the hospital.

### Next Steps

Currently, NeoTree only records data at two clinical time points in KCH: admission and discharge/death, which remains a limitation of the system. Work to expand NeoTree to a daily electronic record is ongoing. Currently, it would be technically feasible to add Days 2 and 3 ward-round forms to the current set of forms, improve longitudinal data collection, and potentially impact the 40% of deaths occurring within 72 h of admission. Implementation experience in SMCH in Zimbabwe has already been reported (23), and NeoTree is now live in a third site (Chinhoyi provincial hospital, Zimbabwe). Next steps include large scale modelling of the causes of neonatal mortality from all NeoTree data, and further refinement and testing of the NeoTree clinical algorithms are in progress. At the moment, NeoTree is running in co-ordination with the Malawi HMIS; however, planned economic evaluation and continued collaboration with Malawi MOH and DHIS teams (as they improve national interoperability) (27) will increase potential for integration and national scale up and reduce duplication of data capture. A modular approach has allowed more progress in linking NeoTree to Zimbabwe's national system as a neonatal module. Ultimately, large-scale rollout and testing, including a cluster randomised control trial to measure impact of NeoTree on the case fatality rate, are planned.

## Conclusion

The NeoTree app and dashboard produced aggregate data, which is likely to be of high quality and reliability, whilst simultaneously providing clinical decision support and quality improvement in a low- resource setting. The dashboard allowed the tracking of monthly trends according to significant events and revealed insights into user behaviour for future refinement of the NeoTree intervention. A large electronic prospective neonatal audit collected at the point-of-care by healthcare professionals on tablet devices was successfully conducted with high data coverage and completeness. Reported neonatal CFR of 204/1,000 may have been slightly lower than the true CFR, but it was still higher than other similar low-resource neonatal units. Key opportunities to improve quality of care were highlighted such as perinatal management of maternal HIV and syphilis and management of out-born babies with hypothermia. This audit represents a key step in establishing real-time electronic audit using systems such as NeoTree in low-resource neonatal facilities in the future.

## Data Availability Statement

The data analysed in this study are locally managed by Kamuzu Central Hospital Malawi. Requests to access these datasets should be directed to msandeni@gmail.com.

## Ethics Statement

The studies involving human participants were reviewed and approved by University of Malawi College of Medicine Research and Ethics Committee (P.02/19/2613) University College London Research Ethics Committee (Ref. 6681/001). Permission to compare data with data collected by the ward clerk was sought from the Malawi PI Dr Chiume, and the Health Management Information System department at KCH. Written informed consent for participation was not required for this study in accordance with the national legislation and the institutional requirements.

## Author Contributions

YM was the chief NeoTree Ambassador leading the implementation of NeoTree in Malawi. She contributed to developing digital forms and dashboard, supervised M&M meetings, controlled the dashboard during the latter months of the study, and wrote the manuscript jointly with the other co-authors. DN contributed to data cleaning, analysed and visualised data, conducted descriptive statistics in R, wrote the results section, and contributed to writing and editing the manuscript. BG and RG wrote the introduction to the manuscript and contributed to editing the final manuscript. FS contributed to visualisations on the dashboard in power BI. TH-B had a project management role, overseeing activities, and supervising NeoTree Ambassadors throughout the audit and approved and edited the final manuscript. ML, FL, and MH supervised CC in her MD thesis. MH is the principal investigator of the NeoTree project, including the Wellcome Trust Digital Innovation award. MC is Malawi the principal investigator for the NeoTree project, including the Wellcome Trust Digital Innovation Award. ML, FL, MH, and MC all contributed to editing the final manuscript. CC conceptualised and supervised the audit study, led the field work in Malawi, and finalised the manuscript. All authors contributed to the article and approved the submitted version.

## Funding

The first 6 months of this study were completed during an MD thesis project of CC, which was funded partly by a clinical fellow post at UCL, the Naughton Cliffe Matthews grant (PI Heys) and partly by charity donations to the NeoTree charity. The second 6 months of the study was supported by the Wellcome Trust (Grant No. 215742_Z_19_Z).

## Conflict of Interest

The authors declare that the research was conducted in the absence of any commercial or financial relationships that could be construed as a potential conflict of interest.

## Publisher's Note

All claims expressed in this article are solely those of the authors and do not necessarily represent those of their affiliated organizations, or those of the publisher, the editors and the reviewers. Any product that may be evaluated in this article, or claim that may be made by its manufacturer, is not guaranteed or endorsed by the publisher.

## Abbreviations

CFR: case fatality rate
HAART: highly active antiretroviral therapy
HIV: human immunodeficiency virus
KCH: Kamuzu Central Hospital
RDS: respiratory distress syndrome
WHO: World Health Organisation
M&M: morbidity and mortality
ZCH: Zomba Central Hospital.
