# Supplementary material for: Admissions to a Low-Resource Neonatal Unit in Malawi Using a Mobile App and Dashboard: A 1-Year Digital Perinatal Outcome Audit
Source: Front Digit Health. 2021 Dec 23;3:761128. doi: 10.3389/fdgth.2021.761128 (PMC8732863; doi:10.3389/fdgth.2021.761128)

Supplementary Material

**Appendix A.** Malawi Ministry of Health Death review form (page 2 on next page).

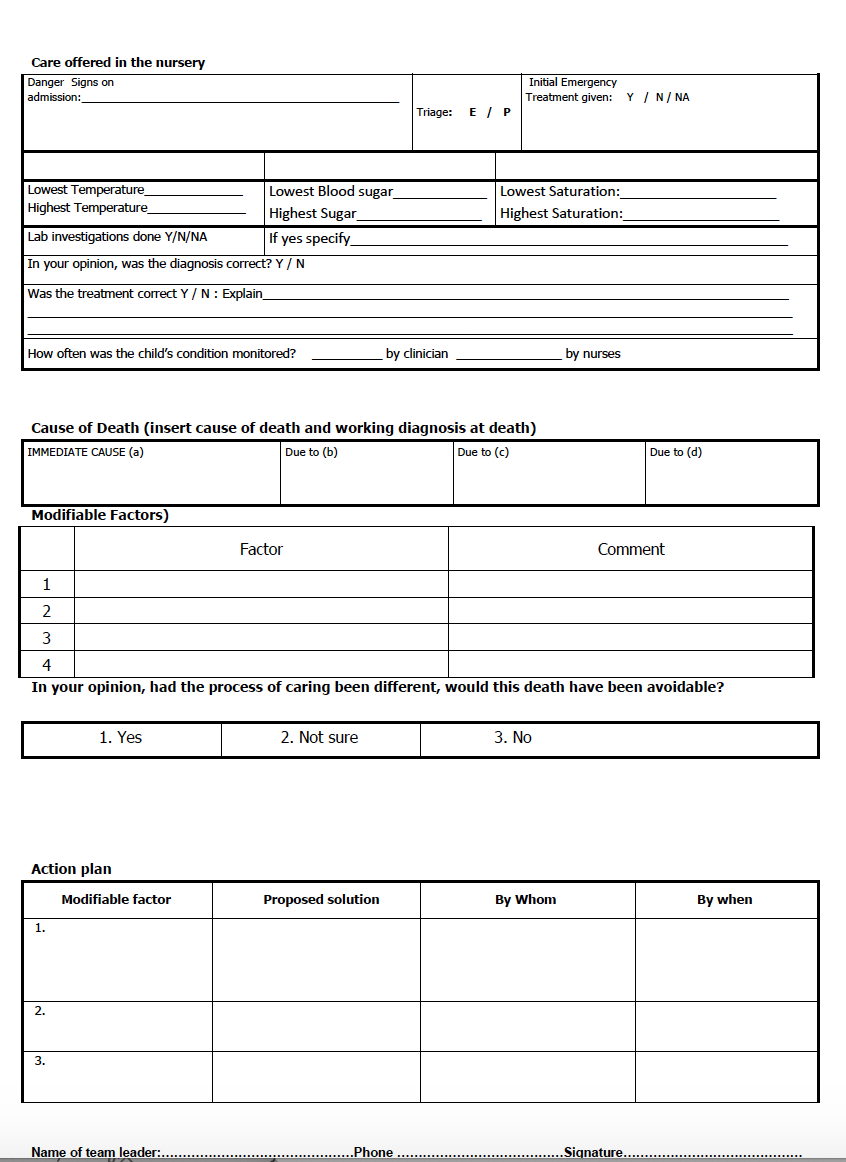


**Appendix B.** Ward logbook / patient register completed by the data clerk at KCH neonatal unit.

**
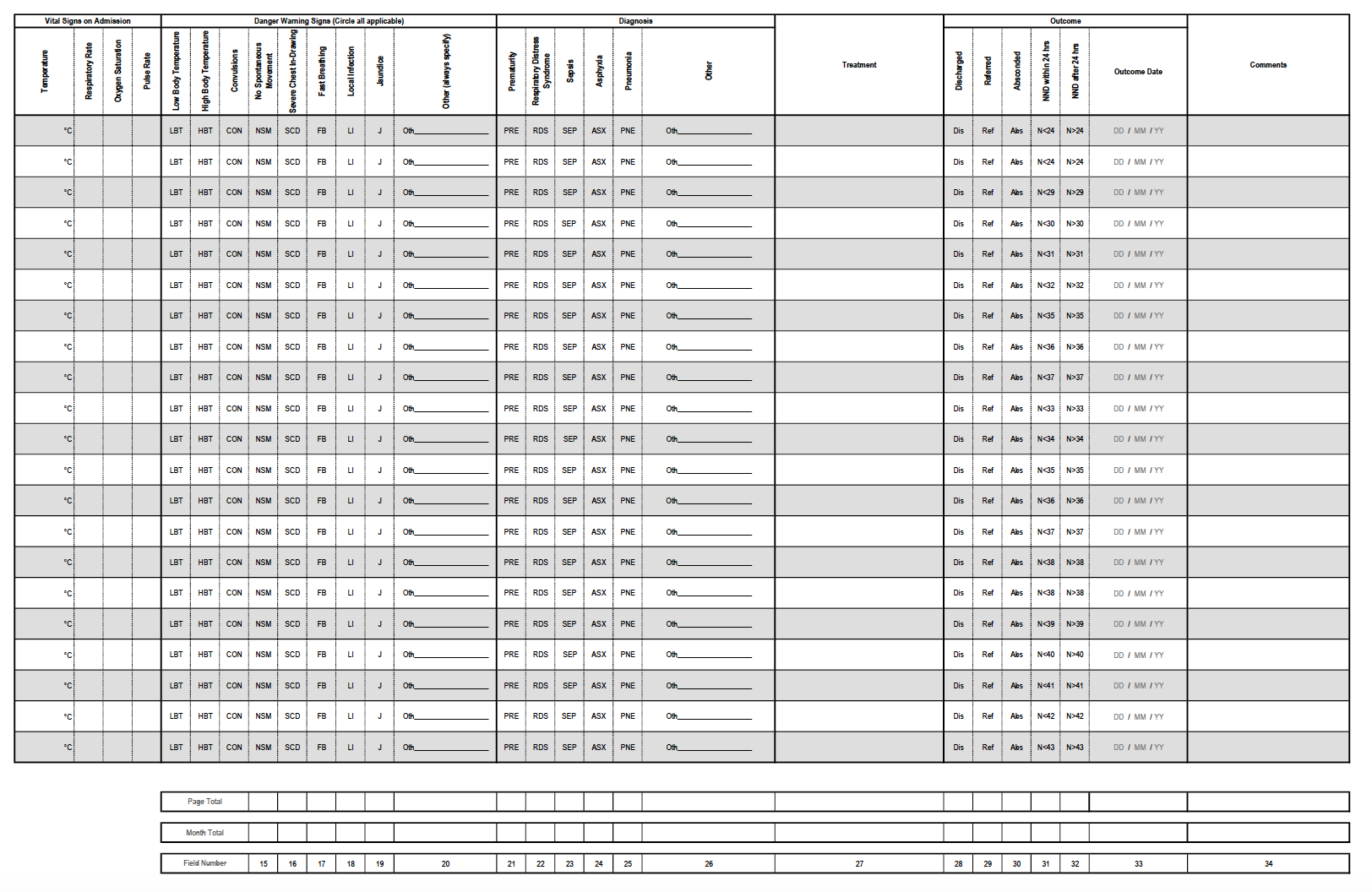
**

**Appendix C.** Aggregate data forms completed by the data clerk each month as part of the usual paper data collection process at KCH neonatal unit.

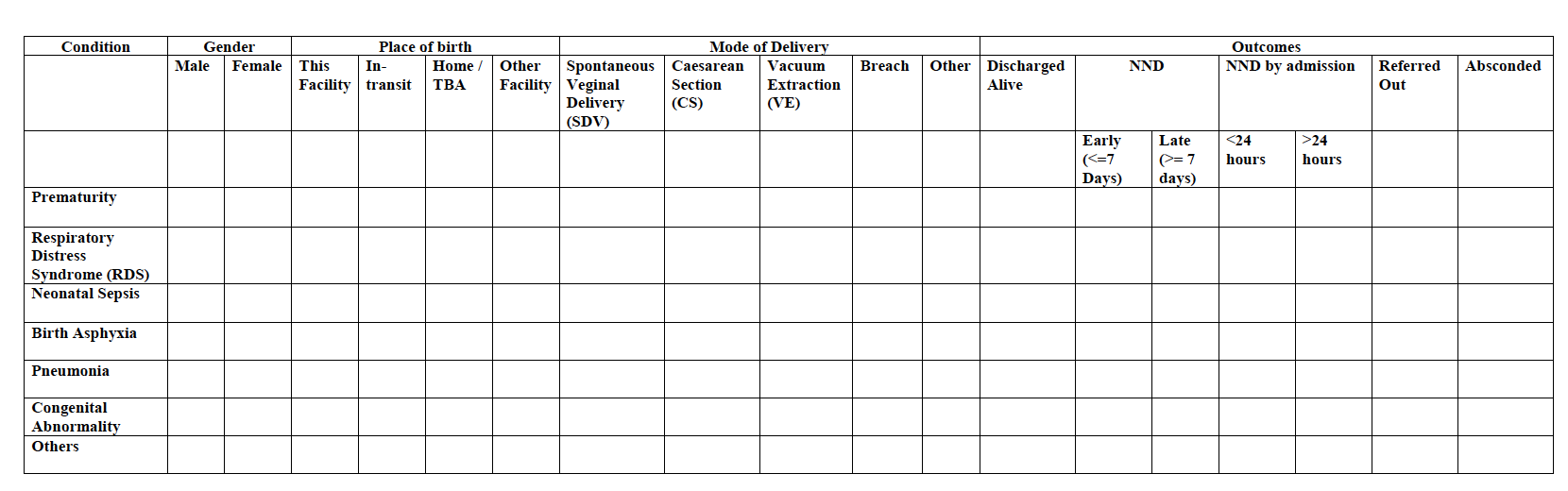

Supplement: Supplementary file 1 [file Data_Sheet_1.docx]
